# Analysis of fungal bloodstream infection in intensive care units in the Meizhou region of China: species distribution and resistance and the risk factors for patient mortality

**DOI:** 10.1186/s12879-020-05291-1

**Published:** 2020-08-14

**Authors:** Guangwen Xiao, Wanqing Liao, Yuenong Zhang, Xiaodong Luo, Cailing Zhang, Guodan Li, Yingping Yang, Yunyao Xu

**Affiliations:** 1 Medical College, Jiaying University, Meizhou, People’s Republic of China; 2 Shanghai Key Laboratory of Medical Fungal Molecular Biology, Shanghai, People’s Republic of China; 3 The First Department of Anesthesiology, People’s Hospital of Meizhou, Meizhou, People’s Republic of China; 4 Department of Anesthesiology, Chinese Medical Hospital of Meizhou, Meizhou, People’s Republic of China; 5 Department of Cardiology, Yuedong Hospital the Third Affiliated Hospital of Sun Yat-Sen University, Meizhou, People’s Republic of China

**Keywords:** Intensive care unit, ICU, Fungal bloodstream infection, Epidemiology, Mortality risk factors

## Abstract

**Background:**

Fungal bloodstream infections (FBI) among intensive care unit (ICU) patients are increasing. Our objective was to characterize the fungal pathogens that cause bloodstream infections and determine the epidemiology and risk factors for patient mortality among ICU patients in Meizhou, China.

**Methods:**

Eighty-one ICU patients with FBI during their stays were included in the study conducted from January 2008 to December 2017. Blood cultures were performed and the antimicrobial susceptibility profiles of the resulting isolates were determined. Logistic multiple regression and ROC curve analysis were used to assess the risk factors for mortality among the cases.

**Results:**

The prevalence of FBI in ICU patients was 0.38% (81/21,098) with a mortality rate of 36% (29/81). Ninety-eight strains of bloodstream-infecting fungi, mainly *Candida* spp., were identified from these patients. *Candida albicans* was most common (43%). Two strains of *C. parapsilosis* were no-sensitive to caspofungin, *C. glabrata* were less than 80% sensitive to azole drugs. Logistic multiple regression showed that age, serum albumin, APACHE II score, three or more underlying diseases, and length of stay in ICU were independent risk factors for mortality in FBI. ROC curve analysis showed that APACHE II scores > 19 and serum albumin ≤25 g/L were the best predictors of mortality.

**Conclusion:**

Candida spp. predominated with high mortality rates among cases of FBI in ICU. Thus, clinical staff should enhance overall patient monitoring and concurrently monitor fungal susceptibility to reduce mortality rates.

## Background

The incidence of fungal bloodstream infections caused by pathogens such as Candida spp. has increased in recent years, especially in intensive care units (ICUs) [1, 2]. Candida bloodstream infections have been reported to be the fourth highest in-hospital infection [3]. Studies have shown that the incidence of fungal bloodstream infections in ICUs is 0.22–4.1% in developing countries [4–7] and 0.024–0.687% in developed countries [8–10]. These rates appear to coincide with more widespread use of broad-spectrum antifungal drugs, glucocorticoids, and immunosuppressive agents, as well as the low immunity of ICU patients and use of central venous catheter technology and other invasive procedures; however, early symptoms of fungal bloodstream infection lack specificity, and low culture-positive rates can lead to a misdiagnosis [11]. Thus, hospitalization and timely, effective treatment might be delayed, increasing costs and mortality risks. In previous research reports, there were several studies on the pathogens and infection risk factors of fungal bloodstream infection and relatively few studies on the risk factors for the mortality of the patients.

Understanding species distribution, resistance, and mortality risk factors associated with pathogenic fungi and ICU fungal bloodstream infections is essential for improving our chances of an early diagnosis, early treatment, and a more positive prognosis. The aim of this study was to analyze the distribution, drug sensitivity, and mortality risk factors of ICU fungal bloodstream infections at three tertiary general hospitals in Meizhou, Guangdong Province, China, to provide baseline reference data for diagnosis and treatment of these infections.

## Methods

### Patient selection

Patients with new infections based on a primary disease who were admitted to ICU of three tertiary hospitals in the Meizhou area were included in the study conducted from January 2008 to December 2017. The patients had failed to respond to antibiotic treatment and tested positive by blood culture for yeast, yeast-like bacteria, and/or mold. We collected basic information on cases, including demographic data (sex, age), disease factors (diabetes, tumors, cardiovascular disease, COPD, digestive tract diseases, urinary system diseases, hypertension, and three or more combined underlying diseases), physiological indicators (serum albumin, serum urea nitrogen, γ--glutamyl peptidase, and APACHE II scores), invasive operational factors (e.g., tracheal intubation, multilumen catheter, central venous catheter, and catheter indwelling time), and treatment factors (e.g., length of ICU stay, gastrointestinal nutrition, emergency catheterization, blood transfusion, central venous catheter, patients in shock receiving catecholamines, and the combined use of antibiotics). The research protocol was approved by the ethics committee of the hospital.

### Culture and laboratory methods

Blood samples that were taken and were cultured using the BACTEC FX400 Automated Blood Culture System (Becton Dickinson, Franklin Lakes, NJ, USA) and the BACTEC Plus/F resin aerobic culture bottle and fungal culture bottle (Becton Dickinson). The VITEK 2-Compact microbial identification and analysis system and yeast biochemical card and analytical profile system systems (bioMérieux, Marcy-l’Étoile, France) were used to identify fungal organisms, using *C. albicans* ATCC 90028 and *C. parapsilosis* ATCC22019 as quality control strains.

ATB FUNGUS 3 fungal susceptibility reagent strips (bioMérieux) were used to determine antimicrobial susceptibilities to caspofungin, amphotericin B, fluconazole, voriconazole, and itraconazole on CHROMagar medium (Zhengzhou Biocell, Henan Province, China), the results were judged according to the current standards of specification.

### Statistical analyses

Data were analyzed using SPSS 21.0 (IBM Corp., Waltham, NY, USA). Variables that displayed a normal distribution were expressed as the mean ± standard deviation (SD) using one-way analysis of variance. Counts were expressed as the number of cases and their percentage rate and significance was measured using the chi-squared test.

Variables that were significant risk factors or close to statistical significance (*P* < 0.1) using univariate analysis were included in a two-class logistic multiple regression model for multivariate survival analysis. The best predictive values of independent risk factors for mortality according to related physiological indicators were analyzed using the ROC, where *P* < 0.05 was considered statistically significant.

## Results

### Prevalence of intensive care unit bloodstream infection and fungal bloodstream infection from 2008 to 2017

The overall prevalence of ICU bloodstream infections was 6.54% (1380/21,098), the highest of which was 7.66% in 2011 and the lowest of which was 5.48% in 2013. The prevalence of fungal bloodstream infections was 0.38% (81/21,098), the highest of which was in 2016 at 0.47% and the lowest of which was in 2008 at 0.26% (Table 1).

**Table 1:** Prevalence of intensive care unit (ICU) fungal bloodstream infection by year from 2008 to 2017

Year	Total blood culture (*n*)	Positive result (*n*/%)	Fungal positive (*n*/%)
2008	1557	112 (7.20%)	4 (0.26%)
2009	1571	109 (6.94%)	5 (0.32%)
2010	1746	113 (6.47%)	6 (0.34%)
2011	1672	128 (7.66%)	6 (0.36%)
2012	1914	121 (6.32%)	8 (0.42%)
2013	2135	117 (5.48%)	7 (0.33%)
2014	2301	158 (6.87%)	10 (0.43%)
2015	2503	147 (5.87%)	10 (0.40%)
2016	2740	190 (6.93%)	13 (0.47%)
2017	2959	185 (6.25%)	12 (0.41%)
Total	21,098	1380 (6.54%)	81 (0.38%)

### Distribution of fungal strains

**Fig. 1:**
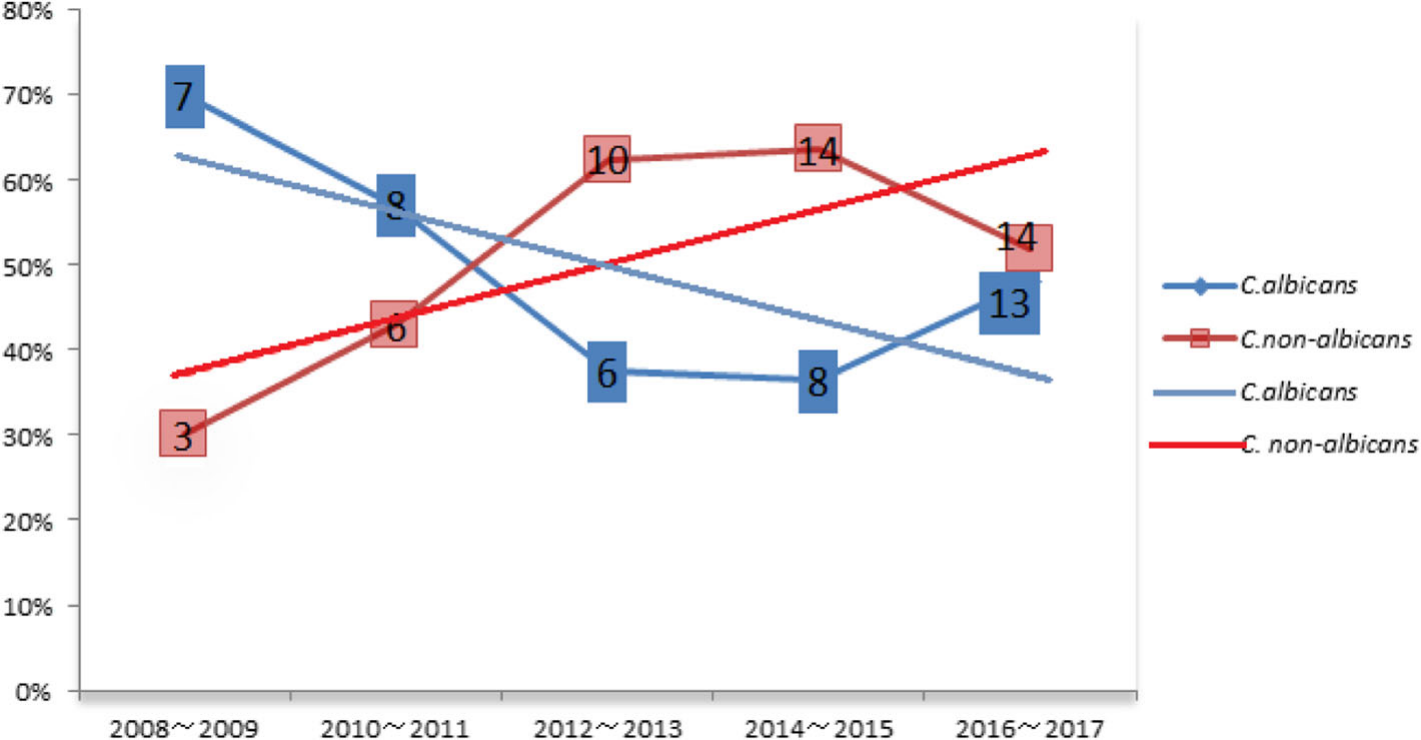
Annual constituent ratio of *Candida albicans* and non-albicans species from fungal bloodstream infections in the intensive care unit (ICU) from 2008 through 2017

Over the 10-year study period, 98 fungal strains from bloodstream infections, mainly *Candida* spp., were obtained from the blood cultures from 81 ICU patients. *Candida albicans* was most common (42/98, 43%), followed by *C. tropicalis* (18/98, 18%), *C. glabrata* (12/98, 12%), *C. parapsilosis* (9/98, 9%), *C. krusei* (5/98, 5%), and *C. guilliermondii* (3/98, 3%). Over the 10-year study period, the prevalence of *C. albicans* decreased, while other Candida spp. increased each year (Fig. 1). In addition, three strains of *Cryptococcus neoformans*, two of *Talaromyces marneffei*, three of *Pichia pastoris*, and one of *Saccharomyces cerevisiae* were isolated.

#### Antimicrobial susceptibility of strains

The drug sensitivity analysis of the five main fungi in the 98 strains showed that > 95% of *C. albicans* strains were sensitive to all antifungal agents tested. All strains of *C. tropicalis* were sensitive to caspofungin and amphotericin B, and > 90% were sensitive to azoles. *Candida glabrata* were relatively less sensitive to azoles; 67% were sensitive to fluconazole and itraconazole, while all strains were sensitive to caspofungin and amphotericin B. Of the *C. parapsilosis* strains, 83% were sensitive to caspofungin, and all were sensitive to the other antifungals (Table 2).

**Table 2:** Susceptibility rates of main fungi to five kinds of antifungal drugs (%)

Fungal species	Number of strains (*n*)	Susceptibility rate (%)				
		caspofungin	Amphotericin B	Fluconazole	Voriconazole	Itraconazole
*Candida albicans*	42	100	97.6	95.2	97.6	100
*Candida tropicalis*	18	100	100	88.8	94.4	94.4
*Candida parapsilosis*	12	83.3	100	100	100	100
*Candida glabrata*	9	100	100	66.6	77.7	66.6

### Risk factor analyses

Twenty-nine of the 81 patients with fungal bloodstream infections in ICU died—a mortality rate of 36%. Univariate analysis of mortality risk factors showed that advanced age, diabetes, cardiovascular disease, lower serum albumin, elevated γ-gamma glutamyl peptidase, APACHE II score, three or more underlying diseases, catheter indwelling time, and ICU stay were associated with the death rates (all *P* < 0.05, Table 3). Sex, malignant tumor, COPD, digestive tract disease, urinary tract disease, hypertension, serum urea nitrogen, tracheal intubation, multilumen catheter, central venous catheter, gastrointestinal nutrition, emergency catheterization, blood transfusion, patients in shock receiving cathecholamines, etc., and combined use of antibiotics were found not to be associated with death from fungal bloodstream infections (Table 3).

**Table 3:** Univariate analysis of mortality risk factors in patients in the intensive care unit with fungal bloodstream infections

Variable	Survival (*n* = 52)	Death (*n* = 29)	χ^2^/F	*P*
Sex, male/female (*n*)	32/20	18/11	0.002	0.962
Age (years; mean ± s.d.)	56.9 ± 10.4	65.7 ± 11.8	10.619	0.001
Disease factors				
Diabetes (*n*)	10	12	4.616	0.032
Malignant tumor (*n*)	3	4	1.518	0.218
Cardiovascular disease (*n*)	8	16	14.135	0.000
Chronic obstructive pulmonary disease (COPD) (*n*)	5	4	0.329	0.566
Digestive tract disease (*n*)	5	4	0.329	0.566
Urinary system disease (*n*)	8	6	0.366	0.545
Hypertension (*n*)	13	13	3.358	0.067
Serum albumin (g/L)	39.5 ± 8.6	33.1 ± 10.7	7.91	0.005
Serum urea nitrogen (μmol/L)	19.1 ± 27	30.7 ± 53.2	1.693	0.193
γ-gamma glutamyl peptidase (U/L)	36.2 ± 24.7	63.8 ± 67.6	6.629	0.010
Acute Physiology and Chronic Health Evaluation (APACHE) II score	16.32 ± 1.74	19.97 ± 2.90	31.565	0.000
Merger of three or more underlying diseases (*n*)	6	12	7.82	0.005
Invasive operative factors				
Tracheal intubation (*n*)	16	13	1.601	0.206
Multi cavity catheter (*n*)	11	11	2.649	0.104
Central venous catheterization (*n*)	23	16	0.893	0.345
Catheter retention time (d)	12.0 ± 4.0	14.6 ± 5.8	5.070	0.024
Therapeutic factors				
Hospitalization time in ICU (d)	9.7 ± 3.8	12.1 ± 6.7	4.401	0.036
Gastrointestinal nutrition (*n*)	21	14	0.472	0.492
Emergency tube (*n*)	18	14	1.454	0.228
Blood transfusion (*n*)	17	12	0.611	0.434
Central venous catheter(*n*)	33	20	0.249	0.804
Patients in shock receiving cathecholamines etc. (*n*)	20	15	1.334	0.350
Combined use of antibiotics (*n*)	25	18	1.464	0.254

χ2/F, chi-squared value; *P* probability

Following multivariate logistic regression, age, low serum albumin, APACHE II score, three or more combined underlying diseases, and ICU time were independently associated with mortality during hospitalization (Table 4).

**Table 4:** Logistic multiple regression analysis of mortality risk factors in patients in the intensive care unit (ICU) with fungal bloodstream infections

Variable	Wald χ^2^	*P*	OR	95% CI
Age	4.380	0.036	1.218	1.013–1.465
Diabetes	3.106	0.078	19.544	0.717–533.069
Cardiovascular disease	0.079	0.779	0.698	0.057–8.610
Hypertension	0.882	0.346	6.494	0.131–322.387
Serum albumin	6.679	0.010	0.639	0.455–0.897
γ-gamma glutamyl peptidase	2.254	0.133	1.047	0.986–1.112
Acute Physiology and Chronic Health Evaluation (APACHE) II score	8.163	0.004	6.330	1.821–22.001
Three or more underlying diseases	4.560	0.030	0.003	0.000–0.576
Catheter retention time	2.607	0.064	1.307	0.984–1.737
Hospitalization time in ICU	4.052	0.035	1.322	1.020–1.714

Wald χ^2^, chi-squared test value; *OR* odds ratio, *CI* confidence interval

### ROC analysis of APACHE II score and serum albumin

**Fig. 2:**
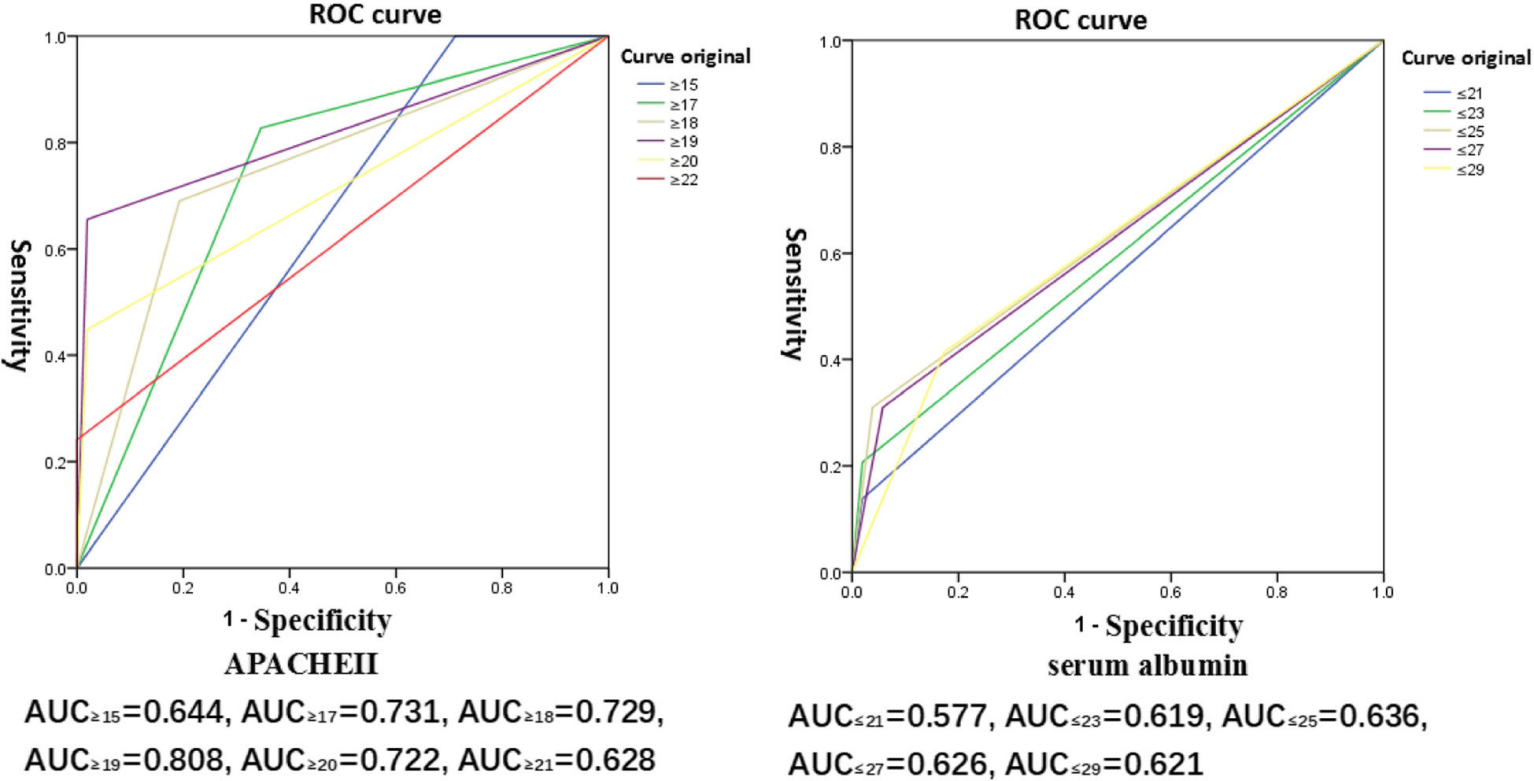
Receiver operation characteristics (ROC) analysis of two independent mortality risk factors—Acute Physiology and Chronic Health Evaluation (APACHE) II score and serum albumin levels

APACHE II score and serum albumin were the factors most strongly associated with mortality among patients with fungal bloodstream infections. After subjecting these variables to ROC analysis, the results showed that when the APACHE II score was > 19, the area under the curve (AUC) was 0.801, and when serum albumin was ≤25 g/L, AUC was 0.636, which indicated that these were the best predictors for mortality among fungal bloodstream infection patients (Fig. 2).

## Discussion

In agreement with our results, previous studies have also shown that the incidence of fungal bloodstream infection and the positive rate of blood culture are increasing each year. This may be closely related to the increasing use of broad-spectrum antibiotics, glucocorticoids, immunosuppressants, radiation therapy, chemotherapy, organ transplantation, catheter surgery, and the emergence of AIDS in recent years [5, 6, 9, 10].

Although the management of invasive fungal infections has made considerable progress in recent years, the prevalence of these infections continues to significantly increase, which warrants close attention from medical care providers. The incidence rate of fungal bloodstream infections in ICU patients in developing countries is 4 to 15 times higher than that in developed countries [11]. In this study, the prevalence of fungal bloodstream infections in ICUs in the three hospitals surveyed in Meizhou from 2008 through 2017 was 0.38%. Compared with fungal bloodstream infections in ICU in developing countries, where the prevalence ranges from 0.22 to 4.1% [4–7], those in Meizhou were low and more comparable to the prevalence among developed countries, where the rates are reported to be 0.024–0.687% [8–10].

*Candida albicans* were more sensitive to azole drugs, such as fluconazole, than other *Candida* spp. Because azole drugs are those most commonly used for clinical fungal treatment, this might explain the increase in the proportion of non-albicans infections. *Candida tropicalis* has a high infection rate in tropical Asia [7, 12]. The Meizhou area is located in the southern part of China and has a subtropical climate, which might contribute to the high rate of ICU fungal infections in that area. Other reports show that *C. parapsilosis* is frequently carried on the hands of medical staff and has a tendency to form a biofilm on medical devices, which might explain its increasing prevalence in the area [13, 14].

In this study, nine patients were also infected with *C. neoformans*, *T. marneffei*, *P. pastoris*, and *S. cerevisiae*. Although the pathogenic ability of *C. neoformans* and *T. marneffei* was strong, that of *P. pastoris* and *S. cerevisiae* was weak. *Pichia pastoris* is used mainly as a research vector and is rarely isolated from human blood.

Most guidelines recommend the use of echinomycin for antifungal infections [15]; however, fluconazole is the most commonly used antifungal drug, and although it has a high mortality rate when used to treat candidemia, it is still widely used in developing countries [16]. In an Indian multicenter study of ICU-acquired candidemia, 64% of the patients were treated with fluconazole [17]. In this study, although most *Candida* spp. were more sensitive to azole drugs, the susceptibility of *C. glabrata* to azole drugs was relatively low, which suggests that testing for antimicrobial susceptibility is necessary for the correct selection of antifungal drugs.

Age is a significant risk factor for nosocomial infections [18], particularly fungal infections of the bloodstream [19, 20]. *Candida parapsilosis* more frequently infects younger populations, while *C. glabrata* and *C. tropicalis* are more common among the elderly [13]. In this study, age was an independent risk factor for infection, and the mortality risk increased with age (Table 4) for several reasons. First, older patients are more likely to have underlying diseases, low immunity, and decreased organ function, which would make them more susceptible to fungal bloodstream infections. Second, older patients might be given fewer antifungal treatments than younger patients, the practice of which is independently associated with a poor prognosis [21]. When the patient’s condition deteriorates, the elderly patient or his or her guardian might decide to stop treatment if the patient’s chance of recovery is low, the costs are higher if treatment is continued, or the patient’s medical insurance does not cover the antifungal drug. Poor care of elderly patients by society and family members, as well as the relatively lagging development of the medical insurance industry, are important problems that affect the health of the elderly in China [22]. To effectively reduce the high mortality rate for elderly patients with fungal disease, measures should be taken to resolve the above-mentioned problems and ensure that antifungal treatment is administered.

Studies have shown that diabetes, tumors, neutropenia, and chronic renal insufficiency are risk factors for fungal bloodstream infections [23, 24]. In this study, diabetes, cardiovascular disease, and three or more underlying diseases were strongly associated with mortality from the infection (Table 3). Logistic multiple regression analysis showed that three or more underlying diseases were among the independent risk factors (Table 4), which might be associated with patients with multiple long-term underlying diseases and many iatrogenic invasive procedures. Treatments such as recent surgery, solid organ transplantation, hemodialysis, longer ICU stay (≥7 d), mechanical ventilation, use of cardiovascular catheters, total parenteral nutrition, and catheters are other suggested risk factors for fungal bloodstream infections [25, 26]. We found that length of stay in the ICU was also an important risk factor for mortality.

The APACHE II score is an important system used to determine the severity of a disease and estimates of mortality and plays an important role in judging the prognosis of bloodstream infections [27]. The higher the APACHE II score, the lower the patient’s immune function; the higher the probability of infecting pathogens, such as fungi; and the higher the chance of death. Serum albumin levels are also important factors that affect bloodstream infections in hospitals [28–30] and are associated with increased mortality from candidemia [12]. Low serum albumin levels affect the body’s immune function, including barrier function, leukocyte phagocytosis, and complement function, resulting in prolonged infection time, anti-infective effects, and increased mortality. Hypoalbuminemia can lead to serious complications, such as sepsis and septic shock [28, 29]. The results of this study showed that the APACHE II score and serum albumin levels were two important independent mortality risk factors in ICU fungal infections (Table 4). According to our findings, we recommend that patients with APACHE II scores > 19 and serum albumin < 25 g/L should receive immediate clinical attention and increased vigilance for possible cases of fungal bloodstream infections.

Our study had several limitations. First, the design of the study limited our access to information on the use of antifungal drugs before hospitalization, and these data could be important for analyzing the emergence of antifungal resistance of the non-albicans species, for example, *C. glabrata*. Second, we analyzed only the presence or absence of exposure to certain risk factors for patient death instead of the duration of exposure. Because the study was not designed to quantify exposure time and the variable was not available for analysis, its associated bias could not be determined. Third, whether patients with fungal bloodstream infections carry AIDS and its related indicators is also an important factor in the death of patients, but in this study, only 2 cases (all deaths) were carried out with AIDS, and fewer cases were not included on analysis of the risk factors for patient mortality. Analysis of risk factors, which may be quite different from studies in other regions. In addition, because of differences in the epidemiological characteristics of antifungal use and candidemia in different countries and regions, we might not be able to extend some of our conclusions to other countries and regions, and additional research is needed in different geographical regions.

## Conclusions

*Candida albicans*, *C. tropicalis*, and *C. parapsilosis* were found to be the most common pathogens that cause fungal bloodstream infections in ICU patients in Meizhou, Guangdong, China. The common fungi in this area are highly sensitive to common antifungal drugs, but attention should be paid to the low sensitivity of *C. glabrata* to azole drugs and the resistance of *C. parapsilosis* to caspofungin. In addition, advanced age, serum albumin, APACHE II score, three or more combined underlying diseases, and ICU stay were independent risk factors for fungal bloodstream infections, each of which must be highly assessed in clinical practice. For high-risk groups, emphasis should also be placed on patient monitoring and risks of infection.

## Data Availability

The datasets used and/or analyzed during the current study are available from the corresponding author on reasonable request.

## Abbreviations

APACHE II: Acute physiology and chronic health evaluation II
ROC: Receiver operating characteristics
COPD: Chronic obstructive pulmonary disease
